# *Kdm6b* Haploinsufficiency Causes ASD/ADHD-Like Behavioral Deficits in Mice

**DOI:** 10.3389/fnbeh.2022.905783

**Published:** 2022-05-31

**Authors:** Yuen Gao, Mohammad B. Aljazi, Jin He

**Affiliations:** Department of Biochemistry and Molecular Biology, College of Natural Science, Michigan State University, East Lansing, MI, United States

**Keywords:** autism spectrum disorder, intellectual disability, attention deficit hyperactivity disorder, KDM6B, haploinsufficiency, mouse model

## Abstract

Autism spectrum disorder (ASD) is a neurodevelopmental disease that has intellectual disability (ID) and attention-deficit/hyperactivity disorder (ADHD) as its common comorbidities. Recent genetic and clinical studies report that *KDM6B*, a gene encoding a histone H3 lysine 27-specific demethylase, is one of the highest ASD risk genes. However, the relationship between *KDM6B* mutations and neurodevelopmental diseases remains unclear. Here we use an animal model to show that genetic deletion of one *Kdm6b* allele in mice leads to autistic-like impaired sociability and object recognition memory. In addition, the mutant mice display markedly increased locomotor activity and impulsivity, two ADHD-like behavioral traits that are ameliorated by methylphenidate treatment. Thus, our study not only uncovers a potential causal link between disruptive *KDM6B* mutations and ASD/ADHD-like behavioral deficits but also provides a new mouse model for studying the cellular and molecular mechanisms underlying the *Kdm6b*-mutation-related neurodevelopmental diseases.

## Introduction

Autism spectrum disorder (ASD) is a clinically heterogenous neurodevelopmental disorder that has impaired sociability and stereotyped behaviors as its core clinical manifestations, and intellectual disability (ID), attention deficit and hyperactivity disorder (ADHD) as its common comorbidities (Park et al., 2016; Hodges et al., 2020; Oberman and Kaufmann, 2020). Earlier studies have revealed that ASD has a strong genetic basis and is associated with various genetic variants (Miles, 2011; Lord et al., 2020). Recent genetic studies on large patient cohorts identify more than one hundred ASD risk genes that have enriched gene functions in chromatin modification and transcriptional regulation (Stessman et al., 2017; Grove et al., 2019; Ruzzo et al., 2019; Lalli et al., 2020; Satterstrom et al., 2020). However, the biological mechanisms underlying the pathogenic roles of identified risk genes in ASD and its comorbid neuropsychological conditions remain largely unknown.

KDM6B is a histone lysine demethylase catalyzing demethylation of tri-methylation of histone H3 lysine 27 (H3K27me3), a transcriptional silencing-related histone modification deposited by Polycomb repressive complex 2 (PRC2; Cao et al., 2002; Agger et al., 2007). Functionally, KDM6B facilitates gene expression through antagonizing the PRC2-mediated gene silencing by erasing histone H3K27me3 at gene promoters (Sen et al., 2008). Previous studies have revealed that KDM6B plays important role in activating the expression of genes involved in neural commitment from pluripotent stem cells, neuronal and glial lineage specification, activity-dependent neuron survival, postnatal neurogenesis, and neuron maturation (Burgold et al., 2008; Park et al., 2014; Wijayatunge et al., 2014, 2018; Shan et al., 2020). Notably, recent genetic studies identify that *KDM6B* is one of the highest ASD risk genes (Satterstrom et al., 2020). The genetic findings are further supported by multiple clinical cases reporting that children diagnosed with neurodevelopmental disorders acquire various disruptive and missense *KDM6B* mutations (Stolerman et al., 2019; Insa Pineda and Gomez Gonzalez, 2021). In addition to ASD behavioral traits, intellectual disability with delayed language and motor skills, cognitive impairment, and ADHD behaviors, patients also display a variety of developmental abnormalities including craniofacial dysmorphism, syndactyly, joint hypermotility, and growth retardation, suggesting critical roles of *KDM6B* in embryonic and postnatal development. However, the pathogenic roles of *KDM6B* mutations in ASD and other comorbid neurodevelopmental conditions remain largely unknown.

In this study, we used the *Kdm6b* knockout (KO) mice to show that *Kdm6b* haploinsufficiency is sufficient to induce the ASD-like deficit in social preference and impaired cognitive memory. Moreover, the heterozygous *Kdm6b*-KO mice show high locomotor activity and impulsivity, two core ADHD-like behavioral deficits that are ameliorated by the methylphenidate treatment. Thus, our behavior-genetics study not only reveals a potential causal link between disruptive *KDM6B* mutations and ASD/ADHD behaviors but also provides a new ASD/ADHD-related animal model for studying the cellular and molecular mechanisms underlying the pathogenesis of *KDM6b*-mutation-associated neurodevelopmental diseases.

## Materials and Methods

### Mice

The sperms of *Kdm6b*-cKO mice were obtained from the Mutant Mouse Resource and Research Centers at UNC (MMRRC; Shpargel et al., 2014). The founder *Kdm6b*-cKO mice were generated *via* *in vitro* fertilization by the MSU transgenic core facility. Mice were housed under standard conditions (12 h light: 12 h dark cycles) with food and water *ad libitum*. The data obtained from all embryos were pooled without discrimination of sexes for the analysis. All mouse experiments were performed with the approval of the Michigan State University Institutional Animal Care & Use Committee.

### Mouse Breeding Strategy

All mice were backcrossed to C57BL/6 mice for at least five generations to reach a pure C57BL/6 background. The heterozygous *Kdm6b-*KO mice (*Kdm6b*^+/1f^) were obtained by crossing the wild-type *Kdm6b*^+/2f^ mice with CMV-Cre mice [B6.C-Tg (CMV-cre) 1Cgn/J, The Jackson Laboratory]. The wild-type (*Kdm6b*^+/+^) and heterozygous *Kdm6b*-KO (*Kdm6b*^+/1f^) mice were generated by *Kdm6b*^+/1f^ × *Kdm6b*^+/1f^ mating.

### Behavioral Tests

All behavioral tests were performed on littermates of wild-type and heterozygous *Kdm6b*-KO mice. The mice were labeled with ear tags. All the behavioral tests were performed by the researcher who was blinded to the genotypes of animals during the behavioral tests. The same animal cohorts were used in the behavioral tests. To minimize the interference effect due to the same experimental condition repeatedly used in consecutive tests, we performed the behavioral tests according to the following order: (1) open field test; (2) novel object recognition test; (3) three-chamber sociability test; (4) marble burying test; (5) elevated plus maze test; and (6) cliff avoidance reaction test. One month after the test (6), open field tests and cliff avoidance reaction tests with saline treatment were performed (7). One week after the test (7), open field tests and cliff avoidance reaction tests with methylphenidate treatment were performed (8).

### Open Field Test

The open field test was used to assess locomotor activity and anxiety-like behaviors in an open field. The method was adapted from the previously reported protocols (Seibenhener and Wooten, 2015; Gao et al., 2021). Mice were acclimated for 60 min to the behavioral testing room before assessment. The open-field apparatus consisted of a custom-made, square white polyvinylchloride foam box (40 × 40 × 40 cm). The test was performed under white lights (~130 lux). To examine the effect of methylphenidate on locomotor activity, saline or methylphenidate was administered 30 min before the open field test. Their behavior was recorded for the first 30 min of habituation to trace and measure time spent in the open field with a digital CCD camera connected to a computer running an automated video tracking software package (ANY-maze, Stoelting Co., Wood Dale, IL, USA). Throughout testing, objects and apparatus were cleaned with 70% ethanol between trials.

### Novel Object Recognition (NOR) Test

The NOR test was used to assess long-term object recognition memory. The protocol used a 3-day paradigm that included habituation, training, and testing as described previously (Ennaceur and Delacour, 1988; Ennaceur et al., 1989; Ennaceur and Meliani, 1992). Mice were acclimated for 60 min to the behavioral testing room before assessment. The testing was performed under red lights to minimize the anxiogenic effect of white light. The behaviors were video recorded and automatically scored using ANY-maze (Stoelting Co., Wood Dale, IL, USA). During habituation (day 1), mice were placed into the open field apparatus, a square white polyvinylchloride foam 40 × 40 × 40 cm box, for 30 min. For training (day 2), two identical objects consisting of blocks were placed in opposite corners of the open field apparatus, and the animals were allowed to explore the objects for 30 min. The object pairs used were counterbalanced across treatments. For testing (day 3), mice were placed in the same apparatus, but this time one object of the pair was replaced with another dissimilar object (novel object), and they were allowed to freely explore for 5 min. Their behavior was recorded, and the time the mice spent with their nose oriented toward the object within 3.5 cm of the object edge was considered exploration time. Throughout testing, objects and apparatus were cleaned with 70% ethanol between trials. The discrimination index was calculated as:


$$DI=\frac{\left(time\;investigating\;novel-time\;investigating\;familiar\right)}{\left(time\;investigating\;novel+time\;investigating\;familiar\right)}$$

### Sociability Test

The three-chamber test was used to assess the sociability of wild-type and *Kdm6b* mutant mice. This test was adapted from Crawley’s sociability and preference for social novelty protocol, which consists of three phases (Moy et al., 2004; Kaidanovich-Beilin et al., 2011). Mice were acclimated for 60 min to the behavioral testing room before testing. The testing was performed under red lights to minimize the anxiogenic effect of white light. The behaviors during all three phases were video recorded and automatically scored using ANY-maze (Stoelting Co., Wood Dale, IL, USA). In phase 1, the experimental mouse was placed in the center of a three-chamber apparatus (polyvinyl chloride, 60 × 40 × 40 cm) and allowed to freely explore for 5 min. During this time, the mouse had free access to all three chambers, which are connected by small openings at the bottom of the dividers. In phase 2, two identical, wire cup-like containers were placed one in each of the side chambers. The experimental mouse was allowed to freely explore the three chambers again for 5 min. In phase 3 (sociability), an unfamiliar same-sex mouse was placed in one of the containers (“social stimulus”), while the other remained empty (“object”). The experimental mouse was allowed to freely explore the three chambers for 5 min. Throughout testing, objects and apparatus were cleaned with 70% ethanol between trials. For analysis, the time with total body spent in each of the three chambers was recorded.

### Marble Burying Test

The marble burying test was used to assess the repetitive behaviors related to anxiety (Deacon, 2006). To perform the marble-burying test, 2-month to 4-month-old mice were placed in a box (27 cm by 15 cm box with 12-cm high walls) with a 7.5 cm depth of bedding for 1 h prior to the test. The mouse was then briefly removed from the testing box and 15 marbles were evenly arranged in a five by three pattern on the surface of the bedding. The mouse was reintroduced into the testing box and was allowed to bury marbles for 10 min. All testing was performed under red lights, and behaviors were video recorded and automatically scored using ANY-maze. At the end of the testing period, the mouse was removed from the box and the number of marbles that were fully buried and partially buried (>50% were buried) were counted.

### Elevated Plus Maze (EPM) Test

The EPM test was used to assess anxiety-like behaviors under stress (Komada et al., 2008). Mice were acclimated for 60 min to the behavioral testing room before assessment. Then the mice are placed in the center of four arms and freely explore for 300 s. All testing was performed under white lights (~130 lux), and behaviors were video recorded and automatically scored using ANY-maze (Stoelting Co., Wood Dale, IL, USA). Throughout testing, the elevated plus maze was cleaned with 70% ethanol between trials.

### Cliff Avoidance Reaction (CAR) Test

The CAR test was used to assess impulsive-like behaviors (Yamashita et al., 2013). The test was initiated by gently placing the animal on a round platform (diameter, 20 cm) supported by a rod (height, 50 cm). The latency from an initial placement on the platform till falling was recorded. Otherwise, the test was continued until 60 min had elapsed. The test was performed under white lights (~130 lux). To examine the effect of methylphenidate on CAR, saline or methylphenidate was administered 30 min before the CAR test.

### Methylphenidate Administration

Saline or methylphenidate hydrochloride (Sigma, M2892) dissolved in saline was intraperitoneally (i.p.) injected at the dose of 2 mg/kg body weight 30 min prior to behavioral tests (Hess et al., 1996).

### Statistical Analysis

All statistical analyses were performed using GraphPad Prism 8 (GraphPad Software). Parametric data were analyzed by a two-tailed *t*-test or two-way ANOVA test for comparisons of multiple samples. *P* values < 0.05 were considered statistically significant. Planned comparisons (Šídák’s multiple comparisons test) were used if ANOVAS showed significant main or interaction effects. Data are presented as mean ± SEM.

## Results

### *Heterozygous Kdm6b* Knockout Mice Display Impaired Sociability

To examine the relationship between disruptive *KDM6B* mutations and ASD genesis, we mated the *Kdm6b* conditional knockout (*Kdm6b*-cKO: *Kdm6b*^+/2f^) mice with CMV-Cre mice to generate a global heterozygous *Kdm6b*-KO (*Kdm6b*^+/1f^) progenies. The heterozygous *Kdm6b*^+/1f^ × heterozygous *Kdm6b*^+/1f^ mating produced a normal number of neonates. Consistent with the previous report (Shpargel et al., 2014), the homozygous *Kdm6b*-KO (*Kdm6b*^1f/1f^) neonates died within 24 h after birth. Therefore, we used wild-type (*Kdm6b*^+/+^) and heterozygous *Kdm6b*-KO (*Kdm6b*^+/1f^) littermates for all behavioral analyses in this study. To exclude any undue effects of strain backgrounds on behaviors, we backcrossed the heterozygous *Kdm6b*-KO mice to the C57BL/6 mice for at least five generations to reach a pure genetic background. We did not observe any obvious differences in postnatal growth or gross body development between wild-type and heterozygous *Kdm6b*-KO littermates.

**Figure 1:**
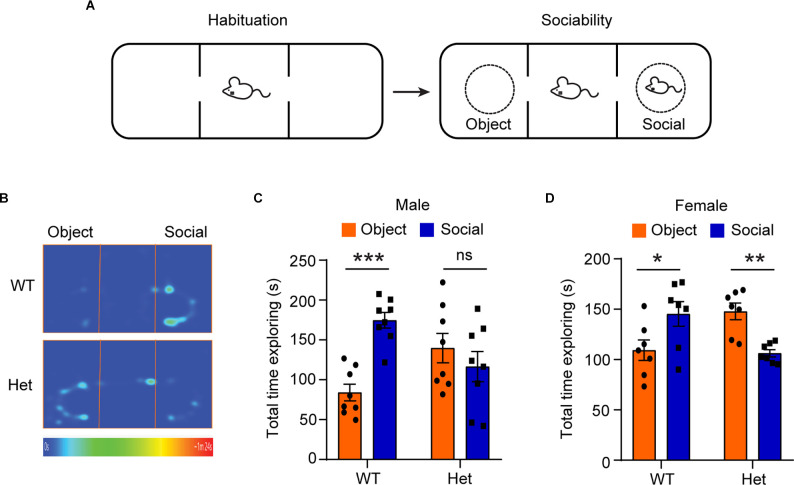
Heterozygous*Kdm6b* knockout mice display impaired sociability.** (A)** Diagram showing the experimental procedure for the sociability test. **(B)** Representative heatmap showing the time that wild-type and heterozygote *Kdm6b*-KO mice spent in exploring the social partner and the non-social object. **(C)** Quantitative results showing the time that male wild-type and heterozygote *Kdm6b*-KO mice spent exploring their social partner and object. *n* = 8 per genotype. P-value calculated using two-way ANOVA test and Sidak’s multiple comparisons test. For the Sidak’s multiple comparisons test: ***p < 0.001, ns: not significant. Error bars in graphs represent mean ± SEM. **(D)** Quantitative results showing the time that female wild-type and heterozygote *Kdm6b*-KO mice spent exploring their social partner and object. *n* = 7 per genotype. P-value calculated using two-way ANOVA test and Sidak’s multiple comparisons test. For the Sidak’s multiple comparisons test: *p < 0.05; **p < 0.01. Error bars in graphs represent mean ± SEM.

To examine whether the heterozygous *Kdm6b*-KO mice develop the core autism-like deficit in sociability, we performed a three-chamber test to examine the voluntary exploration of a social vs. a non-social stimulus (Figure 1A). The results showed main interactive effects of genotype and stimulation type (male: *F_1, 28_* = 14.44, *p* = 0.0007, female: *F_1, 24_* = 17.92, *p* = 0.0003). Planned comparisons revealed that both male and female wild-type controls spent more time with the social stimulus than with the object (male: *p* < 0.001, female: *p* = 0.04), representing normal sociability. In contrast, the heterozygous *Kdm6b*-KO showed either no preference in males or reduced preference in females (male: *p* = 0.39, female: *p* = 0.002) for the social stimulus (Figures 1B–D), suggesting *Kdm6b* haploinsufficiency causes impaired sociability.

### Male Heterozygous *Kdm6b* Knockout Mice Display Impaired Object Recognition Memory

**Figure 2:**
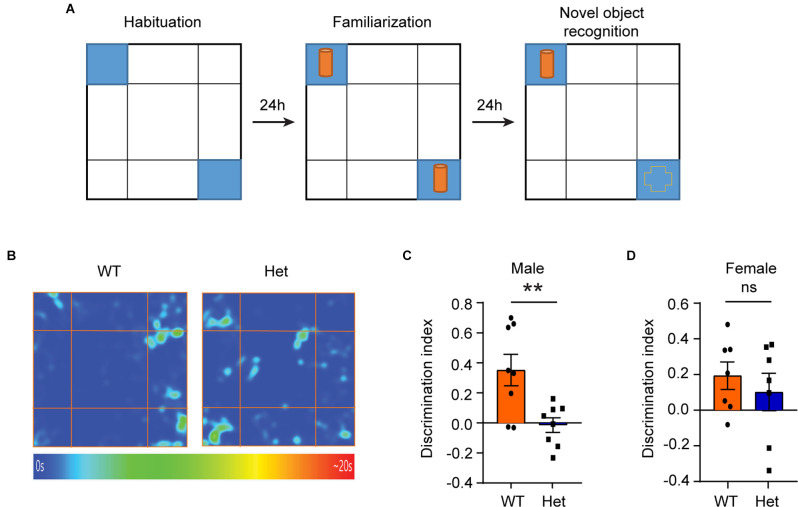
Male heterozygous *Kdm6b* knockout mice display impaired object recognition memory.** (A)** Diagram showing the experimental procedure for the novel object recognition test. **(B)** Representative heatmap showing the time that male wild-type and heterozygote *Kdm6b*-KO mice spent exploring the familiar and novel object. **(C)** Quantitative results showing the NOR discrimination ratio of male mice. *n* = 8 per genotype. P-values calculated using a two-tailed *t*-test. **P < 0.01. Error bars in graphs represent mean ± SEM. **(D)** Quantitative results showing the NOR discrimination ratio of female mice. *n* = 7 per genotype. P-values calculated using a two-tailed *t*-test. ns: not significant. Error bars in graphs represent mean ± SEM.

Besides impaired sociability, some patients with *KDM6B* mutations have language delay, motor-skill delay, and cognitive impairment as their main clinical manifestations (Stolerman et al., 2019). Therefore, we further used the novel object recognition (NOR) test to examine whether the *Kdm6b* haploinsufficiency could lead to impaired object recognition memory. Twenty-four hours after initial familiarization with two identical objects in an arena, the mice were allowed to explore the same arena in the presence of a familiar object and a novel object (Figure 2A). The results showed that compared to wild-type controls, male heterozygous *Kdm6b*-KO mice had a reduced discrimination index favoring the novel object (*t* = 3.173, df = 14, *p* = 0.0068; Figures 2B,C), while the female mutant mice did not show a significant difference in the discrimination index (*t* = 0.7013, df = 12, *p* = 0.4954; Figure 2D), indicating *Kdm6b* haploinsufficiency causes impaired object recognition memory that predominantly affects male mice.

### Heterozygous *Kdm6b* Knockout Mice Display Locomotor Hyperactivity

**Figure 3:**
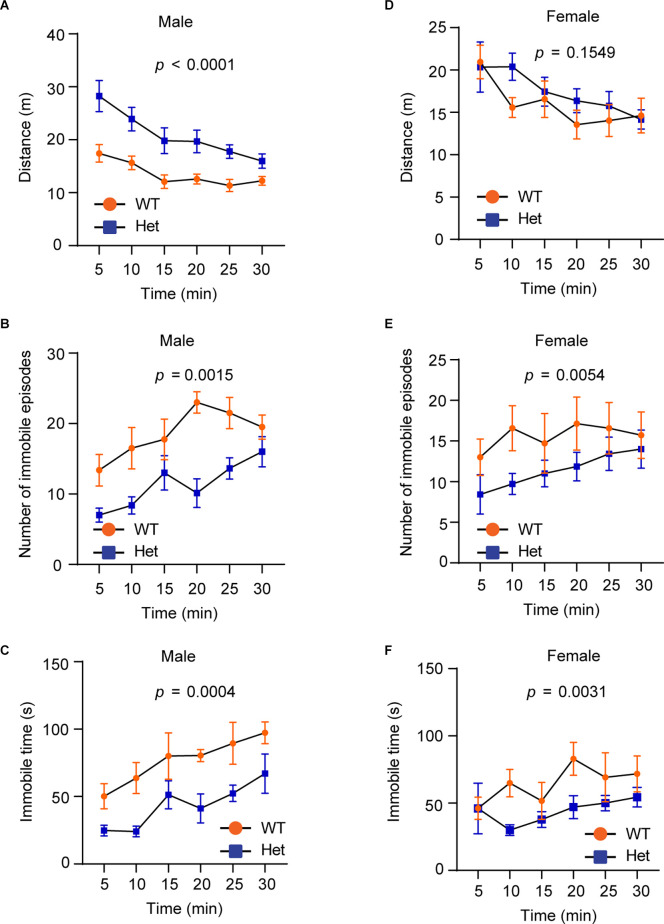
Heterozygous*Kdm6b* knockout mice display locomotor hyperactivity.**(A–C)** The plots showing the running distances**(A)**, immobile episodes **(B)**, and immobile time**(C)** of male wild-type and heterozygous*Kdm6b*-KO mice in a 5-min period for 30 min in theopen-field chamber. P-value calculated usingtwo-way ANOVA test. The results shown are the effect ofgenotype on running distances (*F*_1, 84_ = 53.44,*p* < 0.0001), the effect of genotype on immobile episodes(*F*_1, 84_ = 36.72, *p* = 0.0015), and the effectof genotype on immobile time (*F*_1, 84_ = 29.87, *p* = 0.0004). Error bars in graphs represent mean ± SEM. **(D–F)** The plots showing the running distances **(D)**, immobile episodes **(E)**, and immobile time **(F)** of female wild-type and heterozygous *Kdm6b*-KO mice in a 5-min period for 30 min in the open-field chamber. The results shown are the effect of genotype on running distances (*F*_1, 72_ = 2.066, *p* = 0.1549), the effect of genotype on immobile episodes (*F*_1, 72_ = 8.215, *p* = 0.0054), and the effect of genotype on immobile time (*F*_1, 72_ = 9.340, *p* = 0.0031). Error bars in graphs represent mean ± SEM.

Hyperactivity is a core clinical manifestation observed in ADHD and some ASD patients (Faraone et al., 2015). To examine whether *Kdm6b* haploinsufficiency causes hyperactivity, we performed the open field test to examine the locomotor activity by measuring running distances, immobile episodes, and immobile time in an open field testing chamber. The results showed that although the running distances of wild-type and mutant mice gradually decreased after the first 5 min, male mutant mice still had overall longer running distances in a 30-min period compared to wild-type littermates (*F*_1, 84_ = 53.44, *p* < 0.0001; Figure 3A). Correspondingly, male mutant mice had reduced immobile episodes (*F*_1, 84_ = 36.72, *p* = 0.0015) and time (*F*_1, 84_ = 29.87, *p* = 0.0004) compared to wild-type controls (Figures 3B,C). Different from male mice, female wild-type and mutant mice did not show a significant difference in running distances (*F*_1, 72_ = 2.066, *p* = 0.1549; Figure 3D). However, female mutant mice had significant reduced immobile episodes (*F*_1, 72_ = 8.215, *p* = 0.0054) and immobile time (*F*_1, 72_ = 9.340, *p* = 0.0031; Figures 3E,F). The collective results suggest that *Kdm6b* haploinsufficiency causes locomotor hyperactivity, which appears to affect both sexes although this abnormal behavioral trait is more prominent in male mutant mice.

### Heterozygous *Kdm6b* Knockout Mice Have Reduced Anxiety-Like Behaviors Under Stress

**Figure 4:**
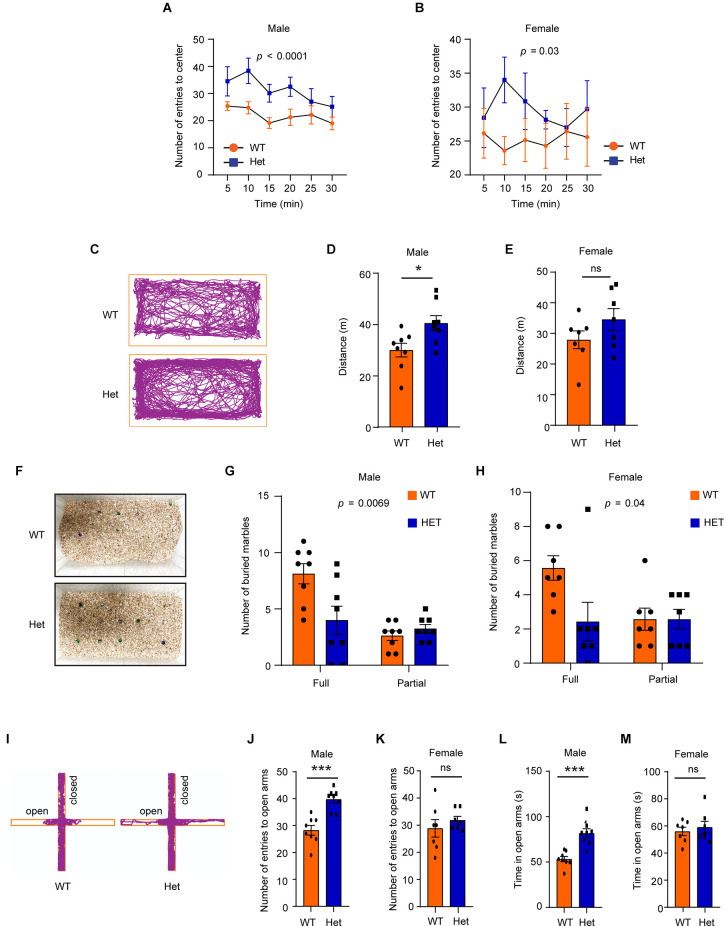
Heterozygous*Kdm6b* knockout mice have reduced anxiety-like behaviors under stress. **(A,B)** The plots showing the number of male **(A)** and female **(B)** wild-type and heterozygous *Kdm6b*-KO mice entering the arena center in a5-min period for 30 min in the open-field chamber. P-value calculated using two-way ANOVA test. The results shown are the effect of genotype on the number of mice entering the arena center (male: *F*_1, 84_ = 21.07, *p* < 0.0001, female: *F*_1, 72_ = 4.89, *p* = 0.03). Error bars in graphs represent mean ± SEM. **(C)** Representative traces of wild-type and heterozygote *Kdm6b*-KO mice in the marble burying testing cages. **(D,E)** The bars showing the running distances of male **(D)** and female **(E)** wild-type and heterozygous *Kdm6b*-KO mice in the marble burying testing cages. *n* = 8 males and 7 females per genotype. P-values calculated using a two-tailed *t*-test. *p < 0.01; ns: not significant. Error bars in graphs represent mean ± SEM. **(F)** Representative photos showing the unburied marbles after marble burying tasks. **(G,H)** The bars showing the numbers of full and partial buried marbles by male **(G)** and female **(H)** wild-type and heterozygous *Kdm6b*-KO mice in the marble burying test. *n* = 8 males and 7 females per genotype. P-value calculated using two-way ANOVA test and Sidak’s multiple comparisons test. The results shown are the effect of genotype on the number of buried marbles (male:*F*_1, 28_ = 8.52, *p* = 0.0069, female:*F*_1, 24_ = 3.88, *p* = 0.04). Error bars in graphs represent mean ± SEM. **(I)** Representative traces of male wild-type and heterozygous *Kdm6b*-KO mice in the open and closed arms of elevated plus maze. **(J,K)** The bars showing the number of male **(J)** and female **(K)** wild-type and heterozygous *Kdm6b*-KO mice entering the open arms in the elevated plus maze. *n* = 8 males and 7 females per genotype. P-values calculated using a two-tailed *t*-test. ***p < 0.001; ns: not significant. Error bars in graphs represent mean ± SEM. **(L,M)** The bars showing the running time of male **(L)** and female **(M)** wild-type and heterozygous *Kdm6b*-KO mice in the open arms of the elevated plus maze. *n* = 8 males and 7 females per genotype. P-values calculated using a two-tailed *t*-test. Note: ***p < 0.001, ns: not significant. Error bars in graphs represent mean ± SEM.

In the open field test, both male and female mutant mice showed a higher number of entries (male: *F*_1, 84_ = 21.07, *p* < 0.0001; female: *F*_1, 72_ = 4.89, *p* = 0.03) into the arena center (Figures 4A,B), suggesting that the mutant mice had lower levels of anxiety-like behaviors under the open-field-induced stress. To further confirm that the heterozygous *Kdm6b*-KO mice had reduced anxiety-like behaviors, we used the marble-burying task, a test reflecting repetitive behavior related to anxiety, to measure the levels of anxiety-like behaviors of wild-type and mutant mice. Consistent with the high locomotor activity observed in the open field test (Figures 3A,D), male but not female *Kdm6b* mutant mice had longer running distances than wild-type controls in home cages (male: *t* = 2.68, df = 14, *p* = 0.017, female: *t* = 1.446, df = 12, *p* = 0.1739; Figures 4C–E). However, compared to wild-type controls, both male and female mutant mice had fewer marbles buried (male: *F*_1, 28_ = 8.52, *p* = 0.0069, female: *F*_1, 24_ = 3.88, *p* = 0.04; Figures 4F–H), suggesting *Kdm6b* haploinsufficiency causes lower levels of anxiety-like behaviors in home cages. Next, we used the elevated plus maze (EPM) test to examine the anxiety-like behaviors under a highly stressful condition by measuring the number of entries into the open arms and the total time for mice running on the open arms. The results showed that compared to wild-type controls, male heterozygous *Kdm6b*-KO mice had a significantly increased number of entries into the open arms (*t* = 5.08, df = 14, *p* = 0.0002) and spent more time in running along the open arms (*t* = 4.93, df = 14, *p* = 0.0002), while wild-type and mutant female mice had a similar number of entries into the open arms (*t* = 0.457, df = 12, *p* = 0.6558) and running time (*t* = 0.5767, df = 12, *p* = 0.5748), suggesting that the *Kdm6b* haploinsufficiency reduced anxiety-like behaviors in male mice under a highly stressful condition (Figures 4I–M). Hence, the collective results suggest that *Kdm6b* haploinsufficiency reduces anxiety-like behaviors under conditions with low or high stress, which appears to prominently affect male mice.

### Heterozygous *Kdm6b* Knockout Mice Display Impulsive-Like Behaviors

**Figure 5:**
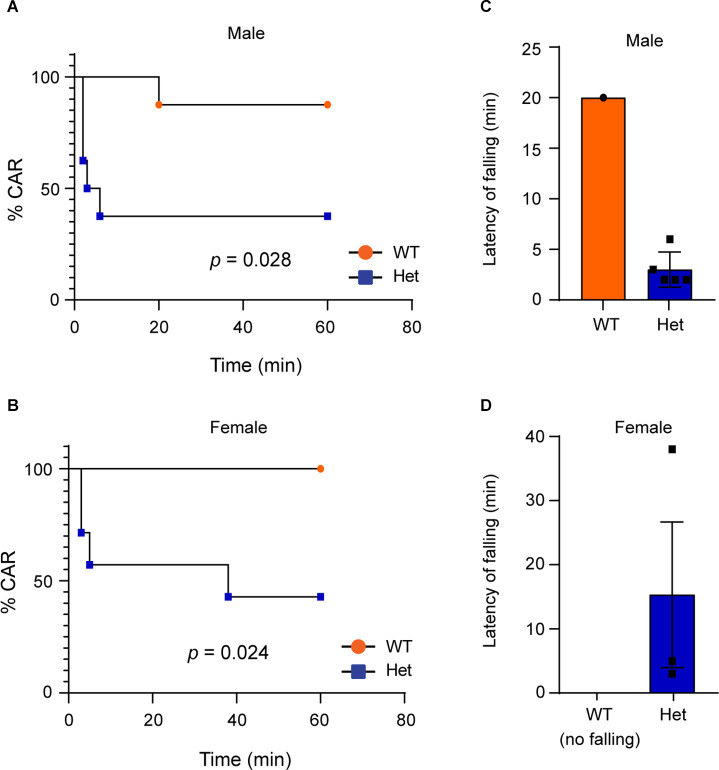
Heterozygous*Kdm6b* knockout mice display impulsive-like behaviors.**(A,B)** Graphs represent the time course of CAR measurement inwild-type and heterozygous *Kdm6b*-cKO male **(A)** and female **(B)** mice in a 60-min CAR testing period. *n* = 8 males and 7 females per genotype. P-values calculated using a Log-rank (Mantel-Cox) test. male: *p* = 0.028; female: *p* = 0.024. **(C,D)** The bars showing the latency of male **(C)** and female **(D)** wild-type and heterozygous *Kdm6b*-KO mice to fall from the platform.

Impulsivity is a main clinical manifestation of ADHD (Faraone et al., 2015). To examine whether *Kdm6b* haploinsufficiency causes impulsive-like behaviors, we used the cliff avoidance reaction (CAR) test to examine whether heterozygous *Kdm6b*-KO mice display impaired CAR by measuring the number as well as the latency for the mice to fall off from an elevated platform. The results showed that compared to wild-type controls, both male and female heterozygous *Kdm6b*-KO had high number of falling from the platform in a 60-min testing period (Mantel-Cox test: male: Chi-square = 4.82, df = 1, *p* = 0.028; female: Chi-square = 5.11, df = 1, *p* = 0.024; Figures 5A,B). Moreover, the latency for heterozygous *Kdm6b*-KO mice (male: 3 ± 1.3 min, mean ± SEM; female: 15.33 ± 10.2 min, mean ± SEM) to fall was much shorter than that of wild-type mice (male: 20 ± 0 min, mean ± SEM; female: no falling; Figures 5C,D). Thus, the results of CAR tests suggest that *Kdm6b* haploinsufficiency causes impulsive-like behaviors in mice.

### Methylphenidate Ameliorates Both Locomotor Hyperactivity and Impulsivity In Heterozygous *Kdm6b* Knockout Mice

**Figure 6:**
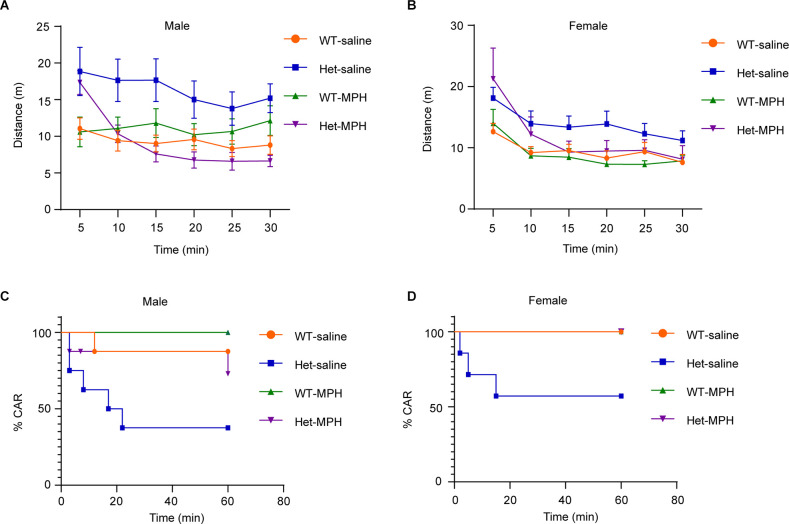
Methylphenidateameliorates both locomotor hyperactivity and impulsivity inheterozygous *Kdm6b* knockout mice. **(A,B)** The plotsshowing the running distances of male **(A)** and female**(B)** wild-type and heterozygous *Kdm6b*-KO micetreated with saline or methylphenidate (MPH) in a 5-min periodfor 30 min in the open-field chamber. For each treatment, *n* = 8 males and 7 females per genotypes. P-value calculated using two-way ANOVA test and Sidak’s multiple comparisons test. The interactive effects of genotype and treatment on running distances: male: *F*_3, 168_ = 19.65, *p* < 0.0001; female: *F*_3, 144_ = 8.154, *p* < 0.0001. *Post-hoc* test: WT-saline vs. Het-saline: male: *p* < 0.0001; female: *p* = 0.0007. WT-MPH vs. Het-MPH: male: *p* = 0.4; female: *p* = 0.12. Error bars in graphs represent mean ± SEM. **(C,D)** Graphs represent the time course of CAR measurement in male **(C)** and female **(D)** wild-type and heterozygous *Kdm6b*-cKO mice treated with saline or MPH in a 60-min CAR testing period. For each treatment, *n* = 8 males and 7 females per genotypes. P-values calculated using a Log-rank (Mantel-Cox) test.The effects of genotype and treatment on CAR, male: Chi square = 10.34, df = 3, *p* = 0.016; female: Chi square = 10.58, df = 3, *p* = 0.014. The effects of genotype in response to treatments, WT-saline vs. Het-saline, male: *p* = 0.0436; female: *p* = 0.048; WT-MPH vs. Het-MPH, male: *p* = 0.13; female: *p* = 0.99.

Hyperactivity and impulsivity are two core behavioral traits of ADHD (Faraone et al., 2015). To further confirm that *Kdm6b* haploinsufficiency-induced locomotor hyperactivity and impulsivity resemble the ADHD behavioral traits, we treated the animals with methylphenidate (MPH), a reagent clinically used to ameliorate ADHD symptoms, to examine whether the hyperactivity and impulsivity observed in mutant mice responded to the MPH treatment. The results showed the main interactive effects of genotype and treatment on running distances (*F*_3, 168_ = 19.65, *p* < 0.0001; female: *F*_3, 144_ = 8.154, *p* < 0.0001). Compared to wild-type controls, *Kdm6b* mutant mice did not respond to the saline treatment and maintained locomotor hyperactivity in the open-field testing chamber (WT-saline vs. Het-saline: male: *p* < 0.0001; female: *p* = 0.0007). In contrast, the *Kdm6b* mutant mice treated with methylphenidate largely reduced their running distances to a similar level as wild-type controls (WT-MPH vs. Het-MPH: male: *p* = 0.4; female: *p* = 0.12; Figures 6A,B). Similarly, CAR tests showed that the effects of genotype and treatment on CAR (male: Chi-square = 10.34, df = 3, *p* = 0.016; female: Chi-square = 10.58, df = 3, *p* = 0.014). Planned comparisons revealed that mutant mice treated with saline still maintained a higher number of falling (WT-saline vs. Het-saline, male: *p* = 0.0436; female: *p* = 0.048). In contrast, methylphenidate treatment ameliorated the CAR of mutant mice by reducing the number of falling to a similar level as wild-type controls (WT-MPH vs. Het-MPH, male: *p* = 0.13; female: *p* = 0.99; Figures 6C,D). Thus, the collective results further suggest that *Kdm6b* haploinsufficiency-induced locomotor hyperactivity and impulsivity resemble the core ADHD behavioral traits observed in human patients.

## Discussion

In this study, we use an animal model to show that genetic deletion of one allele of *Kdm6b*, one of the highest ASD risk genes, is sufficient to induce ASD-like behavioral deficits, suggesting a positive correlation between disruptive *KDM6B* mutations and the core ASD behavioral deficit in sociability. Consistent with the previous report that patients with *KDM6B* mutations have various autistic traits (Stolerman et al., 2019; Insa Pineda and Gomez Gonzalez, 2021), the three chamber test showed that compared to wild-type controls, heterozygous *Kdm6b*-KO mice had impaired sociability (Figure 1), resembling a core social behavioral deficit of ASD. Similarly, the novel object recognition test showed that the mutant mice had impaired long-term object recognition memory (Figure 2), which is consistent with the clinical reports that most patients with *KDM6B* mutations have various degrees of intellectual disability with cognitive impairments (Stolerman et al., 2019; Insa Pineda and Gomez Gonzalez, 2021).

In addition to the deficits in sociability and cognitive memory, ADHD-like behaviors appear to be another prominent phenotype observed in the heterozygous *Kdm6b*-KO mice. Compared to wild-type controls, the mutant mice had a higher locomotor activity with longer running distances, reduced immobile episodes, and decreased immobile time in the open-field testing chamber (Figure 3). Although high locomotor activities of mice could be caused by the anxiogenic effects from the open-field testing chamber and white light used in tests, the locomotor hyperactivity observed in *Kdm6b* mutant mice was unlikely to be caused by increased anxiety since the mutant mice displayed overall lower levels of anxiety-like behaviors in the open-field, marble burying, and elevated plus maze tests (Figure 4). Along with the locomotor hyperactivity, the mutant mice also displayed impaired cliff avoidance reaction, an impulsive-like behavior resembling a core ADHD behavioral trait in patients (Figure 5). Importantly, both locomotor hyperactivity and impulsivity were largely ameliorated by methylphenidate, a reagent clinically used to improve ADHD symptoms (Figure 6). Thus, the collective ADHD-like behavioral deficits, including lower levels of anxiety-like behaviors, locomotor hyperactivity, impulsive-like behaviors, are reminiscent of other ADHD mouse models showing similar phenotypes resembling the core behavioral traits observed in ADHD patients (Wilson, 2000; Zhou et al., 2010).

Although the heterozygous *Kdm6b* mice display a variety of ASD/ADHD-like behavioral traits, it is worth to mention that: (i) the behavioral deficits in male *Kdm6b* mutant mice appear to be more severe than that in female mice, which is consistent with the high prevalence of ASD and ADHD in males (Faraone et al., 2015; Lord et al., 2020); (ii) although the mutant mice do not show typical autistic-like repetitive behaviors like over-grooming, our study does not exclude that the deficits in sociability and object recognition memory in mutant mice could be caused by the possible repetitive nature of behaviors driving the mice to choose familiar and non-social objects in NOR and sociability tests; and (iii) since the locomotor hyperactivity is a prominent behavioral deficit and appears to have a positive correlation with the degrees of impaired sociability and cognitive memory in male and female mutant mice, we do not exclude the possibility that the impairments of sociability and cognitive memory in mutant mice could result from or partially result from hyperactivity. Further identification of the molecular, cellular, and brain mechanisms underlying each behavioral deficit will help to differentiate the roles of ASD-like and ADHD-like traits in contributing to the behavioral phenotypes observed in the *Kdm6b*-KO mice.

Currently, a variety of ADHD rodent models have been developed by genetically targeting putative neural signaling pathways or molecules (Wilson, 2000; Zhou et al., 2010; Leo and Gainetdinov, 2013; Ookubo et al., 2015; Butler et al., 2017; de la Pena et al., 2018). To our knowledge, the heterozygous *Kdm6b*-KO mice reported in this study are the first mice displaying the core ADHD-like behavioral traits that are induced by the deletion of a disease risk gene identified in human patients (Satterstrom et al., 2020). Since the heterozygous *Kdm6b*-KO mice recapitulate a variety of core clinical manifestations observed in patients, they provide an invaluable tool for further exploring the cellular and molecular mechanisms underlying the pathogenesis of *Kdm6b*-mutation-related ASD/ADHD behavioral deficits, as well as for developing and testing new therapeutic approaches to treat neurodevelopmental diseases caused by *Kdm6b* mutations.

## Data Availability Statement

The original contributions presented in the study are included in the article, further inquiries can be directed to the corresponding author.

## Ethics Statement

The animal study was reviewed and approved by Michigan State University Institutional Animal Care & Use Committee.

## Author Contributions

JH conceived the project. YG performed the behavioral tests. MA maintained the mouse colonies. YG, MA, and JH interpreted the data and wrote the manuscript. All authors contributed to the article and approved the submitted version.

## Conflict of Interest

The authors declare that the research was conducted in the absence of any commercial or financial relationships that could be construed as a potential conflict of interest.

## Publisher’s Note

All claims expressed in this article are solely those of the authors and do not necessarily represent those of their affiliated organizations, or those of the publisher, the editors and the reviewers. Any product that may be evaluated in this article, or claim that may be made by its manufacturer, is not guaranteed or endorsed by the publisher.
